# Determination of Bioactive Components in Chinese Herbal Formulae and Pharmacokinetics of Rhein in Rats by UPLC-MS/MS

**DOI:** 10.3390/molecules19044058

**Published:** 2014-04-02

**Authors:** Mei-Ling Hou, Li-Wen Chang, Chi-Hung Lin, Lie-Chwen Lin, Tung-Hu Tsai

**Affiliations:** 1 Institute of Traditional Medicine, School of Medicine, National Yang-Ming University, No. 155, Sec. 2, Li-Nong St, Beitou District, Taipei 11221, Taiwan; E-Mails: maylinghou@gmail.com (M.-L.H.); liwenchang104@gmail.com (L.-W.C.); 2 Institute of Microbiology and Immunology, National Yang-Ming University, No. 155, Sec. 2, Li-Nong St, Beitou District, Taipei 11221, Taiwan; E-Mail: linch@health.gov.tw; 3 National Research Institute of Chinese Medicine, No. 155-1, Sec. 2, Li-Nong St., Beitou District, Taipei 11221, Taiwan; E-Mail: lclin@nricm.edu.tw; 4 Graduate Institute of Acupuncture Science, China Medical University, No. 91, Hsueh-Shih Road, Taichung 404, Taiwan; 5 Department of Education and Research, Taipei City Hospital, No.145, Zhengzhou Rd., Datong Dist., Taipei 103, Taiwan

**Keywords:** herbal analysis, herbal medicine, rhein, pharmacokinetics, liquid chromatography tandem mass spectrometry

## Abstract

Rhein (4,5-dihydroxy-9,10-dioxoanthracene-2-carboxylic acid, cassic acid) is a pharmacological active component found in *Rheum palmatum* L. the major herb of San-Huang-Xie-Xin-Tang (SHXXT), a medicinal herbal product used as a remedy for constipation. Here we have determined multiple bioactive components in SHXXT and investigated the comparative pharmacokinetics of rhein in rats. A sensitive and specific method combining liquid chromatography with electrospray ionization tandem mass spectrometry has been developed and validated to simultaneously quantify six active compounds in the pharmaceutical herbal product SHXXT to further study their pharmacokinetics in rats. Multiple reaction monitoring (MRM) was employed for quantification with switching electrospray ion source polarity between positive and negative modes in a single run. There were no significant matrix effects in the quantitative analysis and the mean recovery for rhein in rat plasma was 91.6% ± 3.4%. The pharmacokinetic data of rhein demonstrate that the herbal formulae or the single herbal extract provide significantly higher absorption rate than the pure compound. This phenomenon suggests that the other herbal ingredients of SHXXT and rhubarb extract significantly enhance the absorption of rhein in rats. In conclusion, the herbal formulae (SHXXT) are more efficient than the single herb (rhubarb) or the pure compound (rhein) in rhein absorption.

## 1. Introduction

Constipation is a common gastrointestinal problem worldwide, and it is estimated that one third of the population of Western industrialized countries suffers from it [1,2,3]. Based on the Rome II diagnostic criteria, constipation affects at least 8.5% of the individuals in Taiwan, occurring more often in females and older persons [4,5]. The aim of treating constipation is to treat the underlying causes, improve symptoms, and resume the normal physiological function of the bowel. Drugs commonly used include bulk laxatives, osmotic laxatives, non-absorbable sugar, stimulant laxatives, cholinergic agents and other prokinetics agents. In Eastern countries, Traditional Chinese Medicine (TCM) is another option in addition to Western medicine for treating constipation [4,6,7]. In many Asian countries, especially in Taiwan, traditional Chinese herbal medicines are frequently prescribed for the treatment many chronic diseases [5,8,9]. According to a survey from a cohort of one million randomly sampled cases from the National Health Insurance database in Taiwan, the Chinese herbal formula San-Huang-Xie-Xin-Tang (SHXXT) is widely used for the treatment of constipation [5]. This herbal formulation consists of rhizomes of *Rheum palmatum* L. (rhubarb), roots of *Scutellaria baicalensis* Georgi and rhizomes of *Coptis* *deltoidea* C. Y. Cheng & P. K. Hsiao, with a weight ratio of 2:1:1, respectively. Generally, based on the ancient process of traditional Chinese medicines preparation, the herbs are mixed and decocted with water before oral administration [10]. However, convenient pharmaceutical herbal products are also used instead of the traditional herbal decocting process. Pharmaceutical herbal products are mainly made by industrial manufacturing methods of decoction, filtration, extraction, concentration, spray or fluid bed granulation, coating and filling [10]. In Taiwan, the ready-to-use pharmaceutical herbal products are frequently prescribed as remedies for chronic diseases [5,8,9]. The alkaloid components, berberine, palmatine, jatrorrhizine and coptisine, are known to be the main effective ingredients isolated from *Coptis* *deltoidea* C. Y. Cheng & P. K. Hsiao; the anthraquinone components, aloe-emodin, rhein and emodin, are the main effective ingredients isolated from *Rheum palmatum* L., and the flavonoid components baicalin, baicalein, wogonoside, and wogonin, are the effective ingredients isolated from *Scutellaria baicalensis* Georgi [11]. Therefore, we chose two representative effective components of each herb to determine the contents of each constituent in different brands of commercial pharmaceutical herbal products and then to investigate the pharmacokinetics of multiple components.

Analytical methods have been reported for the simultaneous determination of different components in Chinese herbal formulae and in functional foods by HPLC-UV (ultraviolet detector) [11,12,13], and HPLC-MS/MS [14,15,16,17]. Furthermore, the assessment of rhein and/or its metabolites from various Chinese herbal formulae in human and animals has been studied [11,17,18]. Although the pharmacokinetics of rhein or Chinese herbal formulae in human and animals has been investigated and determined by HPLC-UV or HPLC-MS/MS, there is still limited information on the comparative pharmacokinetic studies of pure chemicals or single herbs, as well as Chinese herbal formulae.

As *Rheum palmatum* L. is the most common single Chinese herb prescribed for constipation, it is also the major herb in the SHXXT. Most of the studies from the literature have investigated the pharmacokinetics of multiple components in Chinese medicinal herb decoctions which are prepared by mixing all the crude herbs together in a designated ratio, macerating in deionized water, decocting for several hours and then combining each decoction for pharmacokinetic study [11,12,15,17,19]. In our study, we used the convenient ready-to-use pharmaceutical herbal products instead of the traditional herbal decocting process. The products are powder form, that is, their physical and chemical properties are different from those of the decoction form of SHXXT. Thus, the determination of each component that represents each herb is crucial for the further pharmacokinetic study. Second, in contrast to target-oriented Western medicine, Chinese herbal medicine uses a synergistic approach according to the sovereign, minister, assistant and courier principles for constipation treatment. TCMs often consist of a combination of individual herbs to form specific formulae aimed to increase therapeutic efficacy and reduce adverse effects. Although simultaneous determination of different components in Chinese herbal formulae and pharmacokinetic screening of multiple components absorbed in rats have been reported, the information for comparative study concerning the pharmacokinetics of rhein using pure rhein compound, a single Chinese herbal rhubarb (*Rheum palmatum* L.) extract, and the Chinese herbal formula of SHXXT as a pharmaceutical product in rats remain unknown. Hence, the aim of this study is to simultaneously quantify the major biologically active ingredients in the commercial ready-to-use pharmaceutical herbal products by LC-MS/MS methods; the novelty of the study is to investigate the pharmacokinetics of rhein, the pharmaceutical products of rhubarb (*Rheum palmatum* L.) and SHXXT in rats; where the calculated doses of rhubarb and SHXXT are equivalent to those of rhein (11.9 mg/kg).

## 2. Results and Discussion

### 2.1. Optimization of LC-MS/MS Conditions

**Figure 1:**
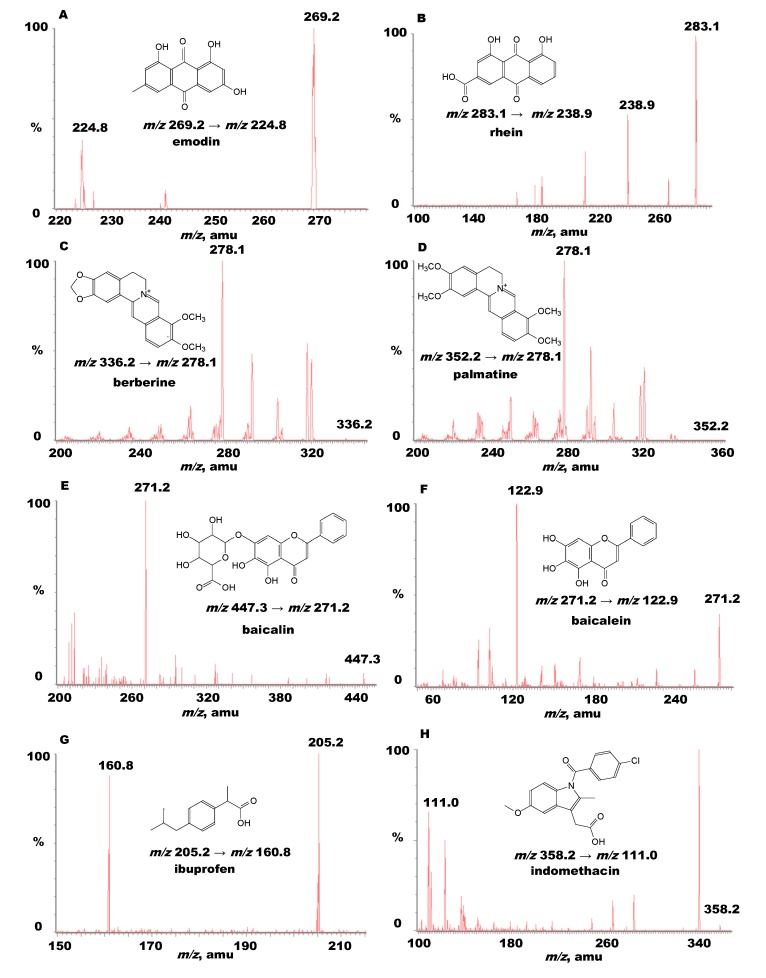
Chemical structures of (**A**) emodin; (**B**) rhein; (**C**) berberine; (**D**) palmatine; (**E**) baicalin; (**F**) baicalein; (**G**) ibuprofen; and (**H**) indomethacin (molecular weight 270, 284, 336, 352, 446, 270, 206, and 357, respectively). Mass spectra of the analytes and their product ions in UPLC-MS/MS with electrospray negative or positive ion mode. The mass transitions of emodin, rhein, berberine, palmatine, baicalin, baicalein, ibuprofen, and indomethacin were m/z 269.2→224.8, 283.1→238.9, 336.2→278.1, 352.2→278.1, 447.3→271.2, 271.2→122.9, 205.2→160.8, 358.2→111.0, respectively.

To optimize the mass spectrometry parameters, a standard solution (100 ng/mL) of emodin, rhein, berberine, palmatine, baicalin, baicalein, ibuprofen, or indomethacin was analyzed by direct injection in the spectrometer. In the analytical condition, the full scan in the positive or negative mode by monitoring the precursor-product combination in the MRM mode was used to identify the analytes. The MRM mode provided high selectivity and sensitivity for the quantification assay (Figure 1). In our study, the validated LC-MS/MS methods were applied for determination of bioactive components in commercial pharmaceutical herbal products and further pharmacokinetic studies, and furthermore, the MRM data demonstrated that the quantitative mass transitions of these analytes are consistent with the previous reports [11,17,18].

**Figure 2:**
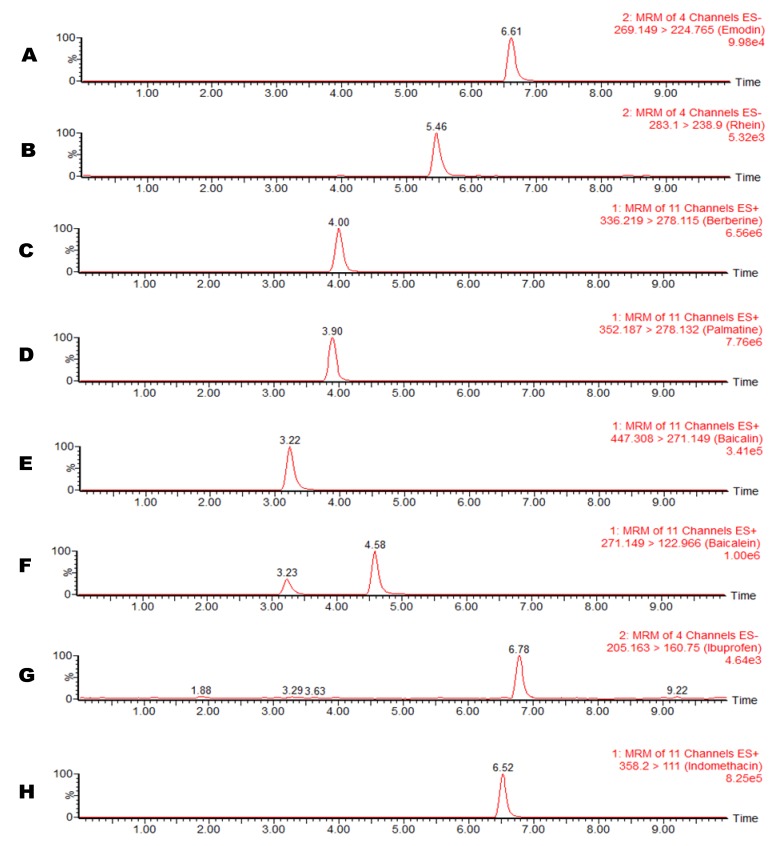
Representative UPLC-MS/MS chromatograms of (**A**) emodin; (**B**) rhein; (**C**) berberine; (**D**) palmatine; (**E**) baicalin; (**F**) baicalein; (**G**) ibuprofen; and (**H**) indomethacin.

After optimizing the detection conditions, the following experiments were conducted to optimize the chromatographic separation of the analytes: chromatographic conditions, especially analytical columns and mobile phase compositions (concentration of buffer, pH value of the buffer and percentage of the organic modifiers), were optimized to achieve good sensitivity and peak shape, as well as a relatively short run. It was observed that acetonitrile gave a better peak shape than methanol, and therefore was selected as the organic phase. A good peak shape could be achieved by adding 0.1% formic acid into the mobile phase. Finally, a mobile phase consisting of acetonitrile- 0.1% formic acid solution (gradient elution) was used in the experiment. The represntative chromatograms of each analyte under the optimized conditions are shown in Figure 2.

### 2.2. Determination of Bioactive Components in Commercial Pharmaceutical Herbal Products

Good linear relationships were obtained for emodin, rhein, berberine, palmatine, baicalin and baicalein, respectively, with a coefficient of estimation r^2^ > 0.995. The intra- and inter-day precision (% RSD) and accuracy (% bias) values of analytes are presented in Table 1, and all % RSD and % bias values were within 15%, showing that the LC-MS/MS method provides excellent quantitative analysis of bioactive components in commercial pharmaceutical herbal products (Table 1). The developed and validated method was applied to analyze seven brands of commercial pharmaceutical herbal products, with each sample extracted and analyzed in quadruplicate. The contents of various constituents in the seven brands of commercial herbal pharmaceutical products are shown in Table 2 and the results demonstrated that although rhubarb is the major herb in SHXXT, anthraquinones such as emodin and rhein were not abundant in SHXXT. The relative abundance of each constituent was as follows: baicalin > berberine > baicalein > palmatine > rhein > emodin. Our quantitative results are consistent with the previous study reported by Zan *et al*. [17,19].

**Table 1:** Intra- and inter-day precision (% RSD) and accuracy (% bias) of the UPLC-MS/MS method for determination of analytes in commercial pharmaceutical herbal products (6 days, six replicates per day).

	Intra-day			Inter-day		
Nominal concentration (ng/mL)	Observed concentration (ng/mL)	Precision (% RSD)	Accuracy (% Bias)	Observed concentration (ng/mL)	Precision (% RSD)	Accuracy (% Bias)
Emodin						
25	25.6 ± 3.84	2.53	14.9	25.7 ± 3.73	2.87	14.5
50	56.2 ± 4.87	12.4	8.67	55.4 ± 4.26	10.8	7.69
100	97.8 ± 2.01	−2.21	2.06	98.4 ± 2.27	−1.64	2.31
250	239 ± 5.31	−4.10	2.22	241 ± 5.63	−3.62	2.33
500	503 ± 3.09	0.65	0.61	504 ± 2.97	0.71	0.59
Rhein						
100	94.1 ± 10.7	−5.86	11.3	103 ± 10.2	3.11	9.11
250	229 ± 24.9	−8.37	10.9	226 ± 25.7	−9.74	11.4
500	492 ± 64.8	−1.56	13.2	506 ± 70.4	1.23	13.9
1000	1017 ± 99.6	1.72	9.79	992 ± 76.3	−0.70	7.68
2500	2480 ± 144	−0.77	5.82	2504 ± 138	0.19	5.51
Berberine						
100	94.3 ± 1.59	1.69	−5.70	97.4 ± 7.26	7.46	−2.60
250	259 ± 2.75	1.06	3.65	252 ± 17.5	6.96	0.67
500	497 ± 0.92	0.19	−0.68	502 ± 13.2	0.62	0.40
Palmatine						
25	28.4 ± 3.48	12.3	13.5	26.2 ± 1.94	7.41	4.88
50	48.5 ± 2.36	4.86	−3.01	48.5 ± 3.06	6.30	−3.07
100	88.1 ± 1.52	1.73	−11.9	92.3 ± 5.69	6.17	−7.74
250	265 ± 8.49	3.20	6.16	265 ± 9.21	3.47	6.00
500	495 ± 3.80	0.77	−1.06	495 ± 3.80	0.77	−1.06
Baicalin						
50	56.3 ± 4.94	8.79	12.6	55.4 ± 4.20	7.59	10.8
100	95.4 ± 4.61	4.83	−4.61	94.3 ± 5.27	5.59	−5.69
250	247 ± 10.6	4.30	−1.40	252 ± 5.05	2.01	0.75
500	503 ± 10.5	2.09	0.51	503 ± 10.4	2.07	0.58
Baicalein						
25	25.0 ± 1.40	5.61	−0.17	25.9 ± 0.99	3.83	3.85
50	53.7 ± 4.62	8.61	7.43	54.8 ± 2.92	5.33	9.61
100	91.3 ± 6.68	7.32	−8.70	92.8 ± 8.48	9.14	−7.17
250	257 ± 18.9	7.40	2.69	249 ± 6.66	2.68	−0.55
500	497 ± 19.9	4.01	−0.52	506 ± 20.9	4.15	1.11
1000	1004 ± 13.7	1.36	0.41	1000 ± 16.6	1.66	−0.01

Data expressed as mean ± SD.

**Table 2:** The contents of various constituents in 7 brands of commercial pharmaceutical herbal products.

Brand	Emodin(mg/g)	Rhein(mg/g)	Berberine(mg/g)	Palmatine(mg/g)	Baicalin(mg/g)	Baicalein(mg/g)
A	0.23 ± 0.01	1.04 ± 0.04	29.2 ± 1.53	2.48 ± 0.08	56.0 ± 6.36	5.26 ± 0.32
B	0.71 ± 0.02	2.38 ± 0.10	26.2 ± 1.60	2.13 ± 0.04	84.1 ± 3.48	16.6 ± 1.00
C	0.39 ± 0.03	0.64 ± 0.06	35.8 ± 2.98	2.72 ± 0.16	36.9 ± 3.48	30.5 ± 1.63
D	0.26 ± 0.01	0.49 ± 0.01	24.1 ± 1.06	2.29 ± 0.08	69.8 ± 4.04	3.69 ± 0.29
E	0.55 ± 0.04	1.07 ± 0.11	28.5 ± 2.20	2.32 ± 0.09	66.4 ± 2.26	5.70 ± 0.46
F	0.07 ± 0.00	0.06 ± 0.02	14.5 ± 0.65	1.11 ± 0.10	32.4 ± 0.81	6.53 ± 0.34
G	0.17 ± 0.01	ND	21.0 ± 2.41	2.05 ± 0.08	18.7 ± 2.25	9.76 ± 2.65
H	1.04 ± 0.04	2.29 ± 0.13	0.01 ± 0.02	ND	0.10 ± 0.00	ND

Seven brands of the commercial Chinese herbal formulae were labeled A-H. H was single Chinese herbs, rhubarb. Data expressed as mean ± SD (*n =* 4). ND, not detected.

According to our quantitative results, although *Scutellaria baicalensis* Georgi is not the major herb in SHXXT, the phenolic compounds, baicalin and baicalein, are predominant (Table 2). Phenolic compounds, ubiquitous in plants, are an essential part of the human diet, and are of considerable interest since they exhibit a wide range of physiological properties [20,21]. These compounds play an important role in growth and reproduction, providing protection against pathogens and predators, besides contributing to the colour and sensory characteristics of fruits and vegetables [20,21,22]. Furthermore, the phenolic contents of plant foods depend on a number of intrinsic and extrinsic factors [20,22]. For example, it is known that the contents of phenolic compounds and composition in wines vary widely and are determined by several factors, such as the variety of grapes used, conditions under which they were grown, wine making techniques, maturity, and processing parameters [21,22]. Hence, the multiple constituents in Chinese herbal formulae make the influence on efficacy of it possible. Natural phenolic compounds have been suggested as ideal substitutes for preservatives in food formulations due to their antioxidant and antimicrobial properties [21]. Thus, phenolic compounds can be alternatively used as functional ingredients to improve the antioxidant capacity of processed foods and to provide the health benefits associated with these phytochemicals [20,21]. According to the physiological properties of phenolic compounds, it is speculated that the predominant phenolic compounds may play the crucial roles in SHXXT.

### 2.3. Method Validation of Rhein in Rat Plasma

**Figure 3:**
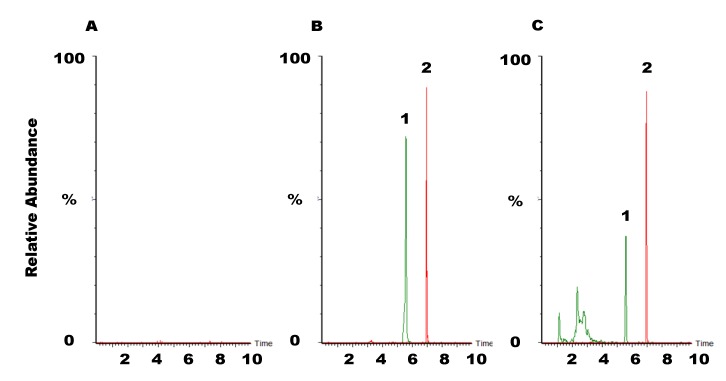
Representative UPLC-MS/MS chromatograms of: (**A**) blank rat plasma sample; (**B**) blank rat plasma sample spiked with rhein (2500 ng/mL) and IS (2500 ng/mL); (**C**) rat plasma sample containing rhein (869 ng/mL) collected at 90 min after SHXXT administration (5 g/kg, p.o.). 1: rhein (retention time: 5.57 min); 2: ibuprofen (IS, 2500 ng/mL, retention time: 6.95 min).

In order to investigate the comparative pharmacokinetics of rhein in rats, a sensitive and reliable analytical method was developed and validated. Each blank plasma sample was tested using protein precipitation procedure and LC-MS/MS conditions to ensure there was no interference of rhein and IS from plasma. The results show that no interferences existed under the present analytical conditions (Figure 3). 

Figure 3A shows the chromatogram of a blank plasma extract with mass transitions of *m/z* 283.1→238.9 for rhein, illustrating a clean baseline with no interference peaks eluted within 10 min. Figure 3B,C show the chromatograms for a standard of rhein and IS (2500 ng/mL, respectively) spiked in blank rat plasma, and for plasma containing rhein (869 ng/mL) collected at 90 min after rhein administration (11.9 mg/kg, p.o.). Each determination was completed within 10 min, and no carry-over peaks were detected in the subsequent chromatograms of plasma samples.

A value of 100% matrix effect indicated that the response in the mobile phase and in the plasma extracts was the same and there was no matrix effect [23]. The matrix effects of rhein and ibuprofen (IS) were 102.9 ± 7.6 and 77.7% ± 4.4% in rat plasma, respectively (Table 3). The mean recoveries for rhein and IS were 91.6 ± 3.4 and 100.7% ± 7.0% in rat plasma, respectively (Table 3).

**Table 3:** Matrix effect and recovery of rhein and ibuprofen in rat plasma.

Nominal concentration (ng/mL)	Set 1	Set 2	Set 3	Matrix effect (%)	Recovery (%)
Rhein					
100	201 ± 25	222 ± 34	211 ± 26	111 ± 17	95 ± 12
500	1464 ± 76	1497 ± 81	1322 ± 105	102 ± 6	88 ± 7
1000	2390 ± 158	2286 ± 157	2085 ± 98	96 ± 7	91 ± 4
Mean ± SD				102.9 ± 7.6	91.6 ± 3.4
Ibuprofen (IS)					
2500	3380 ± 143	2626 ± 147	2646 ± 184	77.7 ± 4.4	100.7 ± 7.0

Data expressed as mean ± SD (*n =* 6). Matrix effect expressed as the ratio of the mean peak area of an analyte spiked post extraction (set 2) to the mean peak area of the same analyte standard (set 1) multiplied by 100. A value of >100% indicates ionization enhancement, and a value of <100% indicates ionization suppression. Recovery calculated as the ratio of the mean peak area of an analyte spiked before extraction (set 3) to the mean peak area of an analyte spiked post extraction (set 2) multiplied by 100.

To evaluate the linearity, calibration standards of six rhein concentration levels from 50 to 2500 ng/mL were prepared and analyzed in blank plasma samples on six different days, six replicates per day. The calibration curve was constructed by plotting the peak-area ratios of rhein to the IS *vs*. the concentrations of rhein. The results demonstrated linearity between the response and the nominal concentration of rhein over the ranges of 50–2,500 ng/mL. The linear regression analysis results showed that the coefficient estimation of the standard curve was >0.995. The data showed excellent reproducibility. The limit of detection (LOD) and quantification (LOQ) of rhein were 50 and 100 ng/mL, respectively. The intra- and inter-day precision (% RSD) and accuracy (% bias) values of rhein in rat plasma are presented in Table 4, and all percent of RSD and bias values were within 15%. These results show that the UPLC-MS/MS method is excellent for the quantitative analysis of rhein in rat plasma extracts.

**Table 4:** Intra- and Inter-day precision (% RSD) and accuracy (% bias) of the UPLC-MS/MS method for determination of rhein in rat plasma (6 days, six replicates per day).

	Intra-day			Inter-day		
Nominal concentration (ng/mL)	Observed concentration (ng/mL)	Precision (% RSD)	Accuracy (% Bias)	Observed concentration (ng/mL)	Precision (% RSD)	Accuracy (% Bias)
Rhein						
100	96.2 ± 9.48	9.86	−3.83	104.4 ± 8.46	8.10	4.39
250	241 ± 20.5	8.51	−3.70	229 ± 20.4	8.93	−8.49
500	567 ± 81.4	14.34	13.43	499 ± 24.1	4.83	−0.20
1000	1057 ± 143	13.50	5.67	1035 ± 129	12.5	3.47
2500	2491 ± 49.7	2.00	−0.36	2505 ± 63.4	2.53	0.20

Data expressed as mean ± SD.

### 2.4. Comparative Pharmacokinetics of Rhein in Rats

Although the pharmacokinetics of SHXXT prepared by decoction and maceration [12,17,19] have been investigated, the pharmacokinetic results demonstrate that different preparation methods affected the pharmacokinetic characteristics of active constituents in SHXXT. Thus, we have investigated the pharmacokinetics of SHXXT preparations from pharmaceutical companies instead of preparing SHXXT decoctions ourselves.

**Figure 4:**
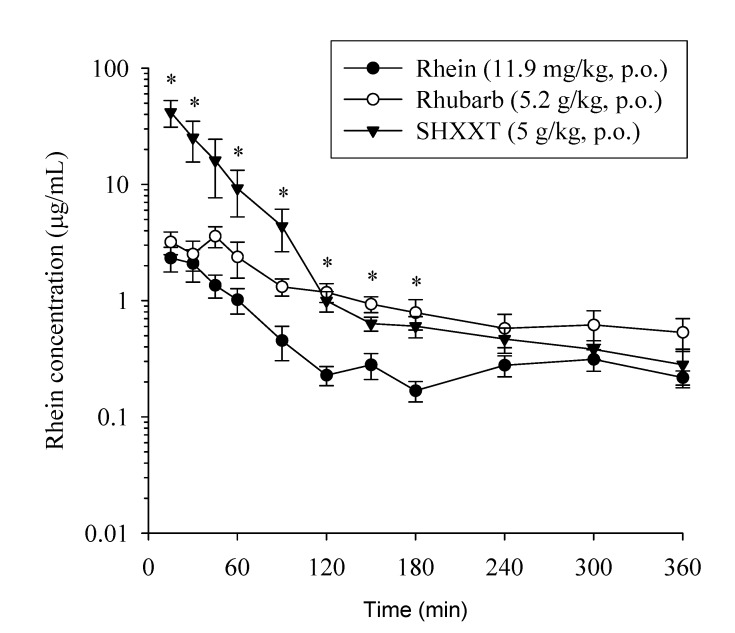
Mean plasma concentration-time profile of rhein after oral dosed with a single Chinese herb (5.2 g/kg, ○), Chinese herbal formulae (5 g/kg, ▼) or rhein (11.9 mg/kg, ●). Each point represents mean ± SEM (*n =* 6). * significantly different from rhein alone at *p <* 0.05.

To compare the pharmacokinetics of rhein as a pure compound, single herbal extract (rhubarb, *Rheum palmatum* L.) and Chinese herbal formulation (SHXXT), the doses of herbal rhubarb and SHXXT extracts were calculated so the doses of rhein were equivalent (11.9 mg/kg). The mean plasma concentration-time profiles of rhein after oral dosage with a single Chinese herb (rhubarb, 5.2 g/kg), Chinese herbal formulae (SHXXT, 5 g/kg) or the pure rhein compound (11.9 mg/kg) to six individual rats for each group are illustrated in Figure 4 and its pharmacokinetic parameters were calculated and are presented in Table 5. As shown in Figure 4, the AUC of rhein in the SHXXT administration group significantly increased by 9-fold (*p <* 0.05) while compared with the rhein administration group. Meanwhile, changes in the pharmacokinetic parameters of rhein in the rhubarb group compared with the rhein administration group were as follows: the T_max_ significantly increased by 190% (*p <* 0.05); however, the total body clearance (CL) significantly reduced by 44% (*p <* 0.05). Moreover, changes in the pharmacokinetic parameters of rhein in the SHXXT group compared with the rhein administration group were as follows: the C_max_ and AUC significantly increased by 17-fold and 9-fold (*p <* 0.05), respectively; however, the elimination half-live, CL, mean residence time (MRT), and volume of distribution (Vss) significantly reduced by 2.9-fold, 6.3-fold, 2.2-fold, and 17.7-fold (*p <* 0.05), respectively.

**Table 5:** Pharmacokinetic parameters of rhein after oral administration of rhein (11.9 mg/kg, p.o.), rhubarb (5.2 g/kg, p.o.), or SHXXT (5 g/kg, p.o.) to six rats.

PK parameters	Rhein	Rhubarb	SHXXT
	11.9 mg/kg	5.2 g/kg	5 g/kg
C_max_ (µg/mL)	2.45 ± 0.55	4.34 ± 0.65	41.8 ± 10.7 *
T_max_ (min)	25 ± 7.42	47.5 ± 7.2 *	15 ± 0
T_1/2_ (min)	144 ± 17.2	179 ± 25.9	50.2 ± 2.73 *
AUC (min µg/mL)	188 ± 36.4	419 ± 80.5	1712 ± 575 *
CL (mL/min/kg)	58 ± 7.89	25.7 ± 4.85 *	9.22 ± 1.44 *
MRT (min)	112 ± 4.29	120 ± 9.12	50.2 ± 4.14 *
Vss (L/kg)	12.2 ± 2.10	6.03 ± 0.97 *	0.69 ± 0.12 *

Data expressed as mean ± SD (*n* = 6). The doses of rhubarb and SHXXT are equivalent to a rhein administration dose of 11.9 mg/kg. Cmax, the peak plasma concentration of a drug after administration; T_max_, the time point of maximum plasma concentration curve; t_1/2_, elimination half-life; AUC, area under the concentration *vs*. time curve; CL, total body clearance; MRT, mean residence time; Vss, volume of distribution. One-way ANOVA followed by Dunnett’s test was used for statistical analysis. * significantly different from rhein alone at *p <* 0.05.

According to our pharmacokinetic study results only rhein existed in the free form, whereas the unconjugated forms of berberine, palmatine, baicalin, baicalein and emodin were not detected after oral administration of SHXXT. It is known that different preparation methods result in significant differences in the pharmacokinetic characteristics of the SHXXT constituents, especially the protoberberine alkaloids [19]; furthermore, the pharmacokinetic results demonstrated that not all the constituents could be detected in rat plasma after oral administration of the SHXXT decoction and maceration. The results of Zhang *et al*. [19] showed that berberine, palmatine, jatrorrhizine and coptisine were detected in the plasma of the decoction group, but were absent in the plasma of the maceration group. Meanwhile, the concentrations of these components in rat plasma after administration of the decoction were very low, with C_max_ under 10 ng/mL [19]. Compared our findings with the previous report of Shia *et al*. [11], indicates that the determination of rhein in plasma may be due to the low glucuronidation activity of UDP-glucuronyltransferases toward the carboxylic acid class of compounds [24]. The absence of berberine and palmatine in the plasma can be explained by an extensive first pass effect since the metabolites of berberine have been determined in human urine and rat plasma after oral administration of berberine [25,26]. The data of Shia *et al*. [11] demonstrated that only the conjugated metabolites of baicalein and emodin were determined in serum, indicating that baicalein and emodin were subject to extensive conjugation metabolism by the intestine and liver during the first pass. Pharmacokinetic studies of rhein in humans [18,27] and rats [11,17,28] have been assessed on the exposure of rhein alone or Chinese herbal formulae contained the ingredient of rhein. These results demonstrated that the linear pharmacokinetics was found for rhein in Chinese healthy subjects after a single oral administration in the range of 50–200 mg. In our study, based on the results of pharmacokinetic parameters of rhein, Chinese herbal formulae containing various ingredients may have an obvious impact on the contribution of a specific chemical to absorption, producing additive, synergetic, or antagonistic effects. Overall, the herbal formulae (SHXXT) are more efficient than the single herb (rhubarb) or the pure compound (rhein) in rhein absorption. Our results demonstrate that the herbal formulae (SHXXT) contains multiple herbs exist synergistic effects based on the basic principle of oriental medicine, the sovereign, minister, assiatant and courier principles.

## 3. Experimental

### 3.1. Chemicals and Reagents

The chemicals emodin, rhein, berberine, palmatine, baicalin, baicalein, ibuprofen and indomethacin were purchased from Sigma-Aldrich Chemicals (St. Louis, MO, USA). LC/MS grade solvents were obtained from J.T. Baker, Inc. (Phillipsburg, NJ, USA) and chromatographic reagents were obtained from Tedia Co., Inc. (Fairfield, OH, USA). Triply deionized water (Millipore, Bedford, MA, USA) was used for all preparations. The pharmaceutical herbal product SHXXT manufactured in accordance with Good Manufacturing Practice (GMP) for Chinese Crude Drugs was obtained from pharmaceutical companies in Taiwan and has been used medicinally for patients. The pharmaceutical herbal product SHXXT were purchased from seven different pharmaceutical manufacturers: Chung Song Zong Pharmaceutical Co., Ltd. (Kaohsiung, Taiwan), Kaiser Pharmaceutical Co., Ltd. (Tainan, Taiwan), Koda Pharmaceutical Co., Ltd. (Taoyuan, Taiwan), Sun Ten Pharmaceutical Co., Ltd. (Taipei, Taiwan), Sheng Chang Pharmaceutical Co., Ltd. (Taipei, Taiwan), Sheng Foong Co., Ltd. (I-Lan, Taiwan), and Wan-Kuo Pharmaceuticals Co., Ltd. (Taipei, Taiwan).

### 3.2. LC-MS/MS

The LC-MS/MS analysis was performed using a Waters Acquity UPLC^TM^ system (Waters, Manchester, UK) consisting of a binary solvent manager, an automatic liquid chromatographic sampler and a Waters Xevo^TM^ tandem quadrupole mass spectrometer equipped with an electrospray ionization (ESI) source. Separation was achieved using a Waters Acquity UPLC type BEH C18 (100 × 2.1 mm, 1.7 µm) analytical column, maintained at 40 °C in a column oven. The mobile phase consisted of A (0.1% formic acid in water) and B (0.1% formic acid in acetonitrile) with a linear gradient elution of 15%–85% (*v/v*) B at 0–8 min, and 15% B at 8–10 min. The flow rate was set at 0.25 mL/min, and the injection volume was 5 µL. For operation in the MS/MS mode, the electrospray ion source was operated with polarity switching between positive and negative ion modes in a single run. The ESI parameters were set as follows: source temperature, 150 °C; desolvation temperature, 500 °C; desolvation gas flow, 800 L/h. The optimized cone voltages (CV) were 48 V for emodin, 49 V for rhein, 15 V for ibuprofen, 46 V for berberine, 40 V for palmatine, 40 V for baicalin, 32 V for baicalein and 27 V for indomethacin. The multiple reaction monitoring (MRM) mode using specific precursor/product ion transitions was employed for quantification. The molecular ions of emodin, rhein, ibuprofen, berberine, palmatine, baicalin, baicalein and indomethacin were fragmented at collision energies of 24, 22, 8, 42, 52, 30, 18 and 45 eV using argon as collision gas. Ion detection was performed by monitoring the transitions: *m/z* 269.2→224.8 for emodin, *m/z* 283.1→238.9 for rhein, *m/z* 205.2→160.8 for ibuprofen, *m/z* 336.2→278.1 for berberine, *m/z* 352.2→278.1 for palmatine, *m/z* 447.3→271.2 for baicalin, *m/z* 271.2→122.9 for baicalein and *m/z* 358.2→111.0 for indomethacin. Ibuprofen was used as the internal standard (IS) for negative ion mode determination, and indomethacin was used as the IS of those four positive ion mode analytes. The software program providing the data platform for spectral acquisition, spectral presentation and peak quantification was the MassLynx 4.1 software package.

### 3.3. Quantitative Determination of Bioactive Components in Commercial Pharmaceutical Chinese Herbal Formulae, Sample Preparation, Method Validation and Sample Analysis

Seven brands of commercial pharmaceutical herbal products of SHXXT, and one brand of a single Chinese herb of rhubarb, were purchased from the seven pharmaceutical manufacturers noted in the above section. All of the commercial pharmaceutical herbal products were labeled as A-H, where H indicates the single Chinese herb, rhubarb. Briefly, an aliquot of 0.1 g precisely weighed SHXXT powder or rhubarb from the above pharmaceutical companies was extracted ultrasonically with 25 mL of 100% methanol for 15 min at room temperature. The extracted solution was centrifuged at 13,000 rpm for 10 min. The supernatant was collected and filtered through a 0.22-µm filter, and the filtrate was analyzed by UPLC-MS/MS. Each sample was extracted and determined in quadruplicate.

All standards were dissolved in methanol to make stock solutions at a concentration of 1.0 mg/mL. Working solutions with mixed analytes were made from each stock solution (emodin, rhein, berberine, palmatine, baicalin, baicalein). The working solutions of the six targeted chemicals were diluted by methanol to construct calibration curves at the designated concentration ranges. Ibuprofen (2,500 ng/mL) and indomethacin (250 ng/mL) were used as the IS for the negative and positive ion mode analytes, respectively. The stock and working solutions were stored at −20 °C. The mass spectra of the six mixed standards and the two IS obtained by UPLC-MS/MS are shown in Figure 1. The calibration curves were plotted based on the ratio of the analyte peak areas to the IS peak areas as a function of the analyte concentration of the standards.

The method validation assays for determination of bioactive components in the pharmaceutical herbal products were carried out according to the currently accepted US Food and Drug Administration (FDA) bioanalytical method validation guidance for specificity, linearity, sensitivity, precision, accuracy, matrix effect and recovery. Standard working solutions were obtained by making appropriate dilutions with IS solutions. All linear curves were required to have a coefficient of estimation of at least >0.995. The intra- and inter-day variability were determined by quantitating six replicates at linear concentrations using the LC-MS/MS method described above on the same day and six consecutive days, respectively. The accuracy (% bias) was calculated from the mean value of observed concentration (C_obs_) and nominal concentration (C_nom_) using the relationship accuracy (% bias) = [(C_obs_ − C_nom_)/C_nom_] × 100. The relative standard deviation (RSD) was calculated from the observed concentrations as precision (% RSD) = [standard deviation (SD)/C_obs_] × 100.

The analyte contents of each pharmaceutical herbal product sample were calculated by the following formula: Content of analyte (mg/g) = (C × V)/M × 10^−6^, where C is the analyte content of the sample, calculated through the regression equation of the IS curve (ng/mL); V is the extraction volume (mL); M is the weight of sample (g).

### 3.4. Method Validation of Rhein in Rat Plasma

The method validation assays for quantification of rhein in rat plasma were carried out according to the currently accepted US Food and Drug Administration (FDA) bioanalytical method validation guidance. The specificity was tested by screening six different batches of drug-free rat plasma for the exclusion of any endogenous co-eluting interference at the peak region of rhein and IS. Each blank sample was tested for interference using the proposed extraction procedure and chromatographic/spectroscopic conditions. The sample preparation for calibration curves was obtained by freshly spiked plasma samples with stock solution of rhein at concentration ranges of 0.05–2.5 µg/mL. A blank plasma sample was also analyzed to confirm absence of interferences. Calibration function was constructed by determining the best-fit of peak area ratios (peak area of rhein/peak area of IS). All linear curves were required to have a coefficient of estimation of at least >0.995. The intra- and inter-day variability were determined by quantitating six replicates at different concentrations using the LC-MS/MS method described above on the same day and six consecutive days, respectively. The precision (% RSD) and accuracy (% bias) of the analytical method were calculated for evaluation. For matrix effect and recovery evaluation, three sets of extraction methods were prepared to evaluate matrix effect and recovery in the quantitative bioanalytical method.

*Set 1*. Standard rhein solutions were constructed using neat solutions of rhein in the mobile phase. The samples were prepared by placing 5 µL of the appropriate concentrations of rhein, and 145 µL of the mobile phase (total volume = 150 µL) into 1.5 mL centrifuge tubes. After mixing, the solutions were transferred into autosampler vials, and 5 µL was injected directly into the LC-MS/MS system.

*Set 2*. Rhein solutions spiked after extraction were constructed in three different lots of plasma by placing 60 µL of plasma in 1.5 mL centrifuge tubes followed by the addition of 120 µL of acetonitrile for protein precipitation. After centrifugation (13,100 *×g* for 10 min, at 4 °C), the supernatant was filtered by a 0.22 µm mini syringe filter. The supernatant (145 µL) was supplemented with 5 µL of appropriate concentrations of rhein. After mixing, the solutions were transferred to autosampler vials, and 5 µL was injected into LC-MS/MS for analysis. In set 2, rhein was spiked after extraction into different lots of plasma, whereas in set 3, rhein was spiked into different lots of plasma before extraction.

*Set 3*. The rhein solutions spiked before extraction were constructed in three different lots of plasma by placing 45 µL of plasma or feces homogenate in 1.5 mL centrifuge tubes to which 5 µL of appropriate concentrations of rhein were added before extraction, followed by the addition of 100 µL of acetonitrile for protein precipitation. After centrifugation (13,100 *×g* for 10 min, at 4 °C), the supernatant was filtered by a 0.22 µm mini syringe filter and was transferred to an autosampler vial, and 5 µL was then injected into LC-MS/MS for analysis.

By comparing the peak areas of the standard rhein solutions, rhein solutions spiked before and after extraction into different lots of plasma, the recovery and ion suppression or enhancement associated with a given lot of plasma were assessed.

Results obtained in this manner were used to determine the matrix effect (ME) and recovery (RE) of the extraction. The peak areas obtained in neat standard solutions in *set 1* were indicated as A, the corresponding peak areas for standard spiked after extraction into plasma or feces homogenate extracts as B (*set 2*), and the areas for standard spiked before extraction as C (*set 3*); the ME and RE values can be calculated as follows:

ME (%) = B/A × 100
RE (%) = C/B × 100

### 3.5. Pharmacokinetic Study of Rhein in Rats

All animal experimental protocols were reviewed and approved by the Institutional Animal Care and Use Committee (IACUC number: 1011202) of National Yang-Ming University. Male specific pathogen-free Sprague-Dawley rats were obtained from the Laboratory Animal Center of the National Yang-Ming University, Taipei, Taiwan. The animals had free access to food (laboratory rodent diet 5P14, PMI Feeds, Richmond, IN, USA) and water. For pharmacokinetic study, Sprague-Dawley rats weighing 220 ± 20 g were anesthetized with pentobarbital (50 mg/kg, i.p.) for cannulation. A polyethylene tube (PE50) was implanted into the left carotid artery for blood sampling. The cannula was exteriorized, fixed in the dorsal region of the neck. Patency of the tube was maintained by flushing with heparinized saline (20 IU/mL). After surgery, the rats were kept in cages individually and allowed to recover for one day.

The dose of herbal formula for translation from human to animal is recommended by the US Food and Drug Administration guidelines as the following conversion equation : Human equivalent dose (HED, mg/kg) = animal dose (mg/kg) × (animal K_m_/human K_m_) [29]. Study on pharmacokinetics of rhein at 50, 100, and 200 mg in human (60 kg in adults) has been investigated [18], and therefore, rhein at 11.9 mg/kg was studied in this study according to the dose translation equation. Briefly, the pure compound rhein, the rhubarb extract, and the SHXXT formula suspended in water at doses of 11.9 mg/kg, 5.2 g/kg, and 5 g/kg were individually administered to rats by oral gavage. The doses of rhubarb extract and SHXXT extract were equivalent to rhein administration dose of 11.9 mg/kg. About 200 µL of blood samples was withdrawn from the cannula implanted in the carotid artery and placed into a heparin-rinsed vial at 0, 15, 30, 45, 60, 90, 120, 150, 180, 240, 300 and 360 min. The method of plasma sample preparation was slightly modified from the previous study [30]. For quantitative analysis, the plasma sample (50 µL) was vortex-mixed with acetonitrile (100 µL) for protein precipitation. Data from these samples were used to construct the pharmacokinetic curve of rhein. The plasma sample was diluted by blank plasma sample at an appropriate ratio before analysis if the rhein concentration was excess 2500 ng/mL.

Pharmacokinetic calculations were performed on each individual set of data using the pharmacokinetic software WinNonlin Standard Edition, version 1.1 (Pharsight Corp., Mountain View, CA, USA) by noncompartmental methods.

### 3.6. Statistical Analysis

Data are expressed as the mean ± SD or mean ± SEM. Comparison among groups were performed using one-way analysis of variance (ANOVA) followed by Dunnett’s test, and differences were considered statistically significant when *p* < 0.05.

## 4. Conclusions

An UPLC-MS/MS method for the determination and quantification of bioactive components in commercial pharmaceutical Chinese herbal products, and its application to determine the comparative pharmacokinetics of rhein in rat plasma have been developed and validated. The method was demonstrated to be selective, precise, accurate, and reliable for the simultaneous determination of various constituents present in this complex herbal prescription, and for quantification of rhein in biological samples. In addition, the method was applied for a pharmacokinetic study in freely moving rats. The comparative pharmacokinetic parameters of rhein in rats after oral dosage with the pure compound (rhein), the single herb (rhubarb), or the Chinese herbal formulae (SHXXT) are here reported for the first time. These pharmacokinetic parameters may be used as reference for the clinical prescription compatibility of traditional Chinese medicine prescriptions. 
